# Medico-Legal Applications of the Human Microbiome and Critical Issues Due to Environmental Transfer: A Review

**DOI:** 10.3390/microorganisms12122424

**Published:** 2024-11-25

**Authors:** Giulia Ricchezze, Erika Buratti, Francesco De Micco, Mariano Cingolani, Roberto Scendoni

**Affiliations:** 1 Department of Law, Institute of Legal Medicine, University of Macerata, 62100 Macerata, Italy; e.buratti1@unimc.it (E.B.); mariano.cingolani@unimc.it (M.C.); r.scendoni@unimc.it (R.S.); 2 Research Unit of Bioethics and Humanities, Department of Medicine and Surgery, Università Campus Bio-Medico di Roma, 00128 Roma, Italy; f.demicco@unicampus.it

**Keywords:** microbiome, transfer, environment, forensics

## Abstract

Microbiome has recently seen an increase in its forensic applications. It could be employed to identify a suspect when DNA is not available; it can be used to establish postmortem interval (PMI). Furthermore, it could prove to be fundamental in cases of sexual assault. One of the most interesting aspects to study is how microbiomes are transferred. The aim of this review is to analyze the existing literature focusing on the potential transfer of microbiome from humans to environment. Searches on PubMed, Scopus, and Web of Science identified a total of 348 articles. Furthermore, from the bibliographies of the included articles, an additional publication was selected, in accordance with the established inclusion and exclusion criteria. This study has shown the potential of utilizing microbiomes as trace evidence, particularly in connecting individuals to specific environments or objects. However, the variability and dynamics of microbial transfer and persistence need to be carefully addressed.

## 1. Introduction

The term human microbiome refers to the full set of genome sequences found within the ecological community of microorganisms—commensal, symbiotic, and pathogenic—that coexist within a person’s body. This community, known as the microbiota, includes bacteria, fungi, protozoa, and viruses [1]. It comprises 10 to 100 trillion symbiotic microbial cells, unique to each individual. In Sender et al.’s model of a “reference man” (aged 20–30 years, weighing 70 kg, and standing 170 cm tall), the estimated total number of microbial cells is 3.8 × 10^13^, with a combined mass of 0.2 kg [2,3]. The human microbiome is extremely variable depending on its functional role in a particular body site; we therefore talk about the microbiomes of the skin, intestinal cavity, oral cavity, respiratory tract, hair, etc.

In recent years there has been a surge in microbiome research in medicine, general healthcare, and biology [4]. Today, the study of the microbiome for forensic purposes is a fast-growing field with important modern applications [5,6]. Indeed, some studies [7] have demonstrated the enormous potential of microbiome analysis as a tool for forensic identification, especially since forensic work is often hampered by degraded or inadequate DNA samples. A human microbiome contains over one million genes—about 500 times the number found in the human genome. The composition of an individual’s microbial communities is shaped by genetic factors as well as by environmental influences and lifestyle choices, such as diet, sleep habits, smoking, and alcohol use [8,9,10]. In theory, each person carries a distinct set of microorganisms that can be identified through microbiome analysis [11]. Thus, the sequencing of an individual’s microbiome could help to develop leads in criminal investigations. It could also assist in identifying a suspect based on microbiome samples retrieved from the crime scene or directly from the suspect or the victim.

Additionally, gaining a deeper understanding of microbiome transmission between individuals could aid in investigating cases of sexual assault [12,13]. Ghemrawi et al. examined genital microbial signatures by analyzing ten genital samples (five from males and five from females) and compared their findings with data from longitudinal studies [14].

Microbiome analysis has also shown promise in other forensic contexts [15]. For instance, Pechal et al. [16] studied the thanatomicrobiome as an indicator of ante-mortem health, potentially enriching the biological profile. They analyzed 83 microbial taxonomic profiles (collected within 24 h postmortem), categorizing them into two groups: cases with autopsy-confirmed heart disease and cases involving violent death. Heart disease evidence was based on heart examination, including microscopic analysis, along with medical history. To explore potential statistical links between the postmortem microbiome and ante-mortem health, the researchers applied binomial logistic regression to compare microbial diversity with heart disease presence. In particular, they observed phylogenetic diversity in the oral bacterial community in cases with heart disease.

The Earth Microbiome Project (EMP) was launched in 2010 to explore global biogeographical variations and the factors influencing them, such as climate, altitude, latitude, and soil characteristics. Understanding microbial diversity could offer insights into an individual’s geographical origin [17]. This could be an important request in the field of forensic geolocation [18].

Considering that the microbiomes responsible for mammalian decomposition are consistent and reproducible across various hosts and environments, studying the microbiome can also offer applications for postmortem interval (PMI) estimation [19,20]. The most strictly controlled experiments have been performed on animals, although studies on human cadavers have also been published recently, despite the limited number of human cadaver samples [21,22]. A huge population of cutaneous microbiota live on human skin and can be effectively transferred to many surfaces or items. These microbial networks can remain on surfaces for a significant amount of time since they have high protection from ecological pressures such as dampness, temperature, and bright radiation [23].

This review aims to explore the existing literature on the potential transfer of the human microbiome to the environment. This aspect has a fundamental importance since environmental contamination could compromise any evidence retrieved at any crime scene or other medico-legal contexts.

## 2. Materials and Methods

This systematic review was conducted in accordance with the PRISMA (Preferred Reporting Items for Systematic Reviews and Meta-Analyses) reporting guidelines [24]. The PRISMA checklist is available in Supplementary Materials Table S1.

A systematic literature search was performed using PubMed, Scopus, and Web of Science to find studies published in English.

The goal of this study was to review the existing literature on microbiome transfer. This included outlining the research methodology, identifying the types of studies that have incorporated genetic analysis, and evaluating their practical applications and effectiveness in autopsy procedures, as summarized from the available literature.

The generic free-text search terms were: (“Microbiome” [All Fields]) AND (“transfer” [All Fields]) AND (“environment” [All Fields]) AND (“forensics”).

Two researchers conducted independent searches of PubMed, Scopus, and Web of Science for relevant studies, while a third researcher verified that the selected articles met the inclusion criteria. The following data were extracted from the chosen studies: authors, country of study, publication date, number of samples analyzed, and mode of transfer. The resulting documents were further refined by reviewing their language, title, abstract, methods, and keywords. Ultimately, the studies selected for analysis had to meet the following inclusion criteria:

- Original articles or case studies;
- Studies analyzing if microbiome transfers.

Non-inclusion and exclusion criteria were:

- Studies focusing on making clinical diagnosis;
- Animal studies;
- Studies on microbiome patterns without transferring;
- Reviews.

**Figure 1:**
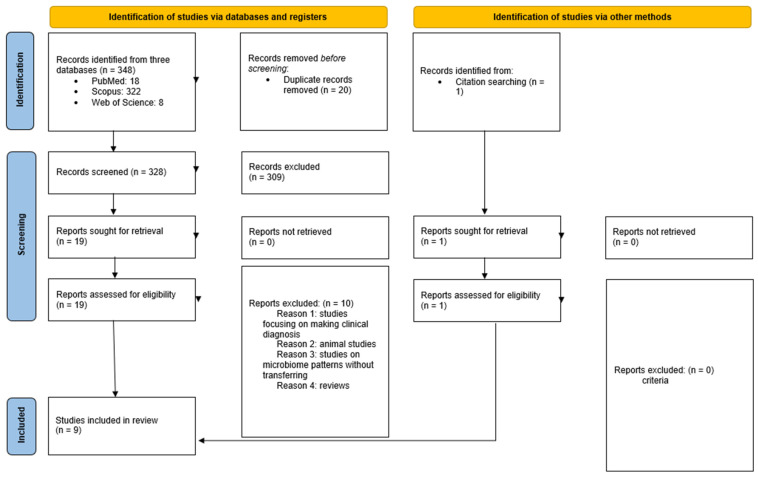
Descriptive diagram of the paper selection process.

The study was designed according to the PRISMA recommendations, as shown in Figure 1.

As previously noted, three researchers independently evaluated whether the articles’ titles or abstracts met the inclusion criteria, resolving any disagreements through consensus. One researcher conducted the data extraction, which was then reviewed by another researcher and further validated by an additional pair of investigators.

## 3. Results

In total, 348 publications met the search criteria. We excluded 20 duplicate articles. After thorough evaluation against the primary inclusion criteria, an additional 309 publications were excluded, resulting in 19 full-text articles. Only those publications that examined the transfer of the microbiome were selected, leading to the exclusion of another 10 articles from the sample. Consequently, nine full-text articles fully met the inclusion criteria for the review. The article selection process is illustrated in Figure 1.

Of these nine articles, eight studies were conducted on volunteers and/or environments, while only one article involved a study conducted on human cadavers.

Regarding the country of the included studies, two were performed in the United Kingdom jointly to Italy, three in Australia, one in China, and three in the United States. Specifically, the only included study conducted on cadavers was performed in the United States, given the more favorable laws that allow the use of human cadavers for scientific purposes. Table 1 summarizes the main findings of each article.

## 4. Discussion

This review highlights the potential of microbiome analysis in forensic science, particularly regarding the transfer and persistence of skin-associated microbiomes on textiles, surfaces, and personal protective equipment.

Before discussing the results obtained, it is useful to briefly describe what the main techniques used in microbiome analysis are. Two widely used techniques are deep sequence surveys of PCR-amplified marker genes (such as 16S rRNA) and whole-genome shotgun metagenomics, where the entire microbial DNA in a sample is sequenced [34]. However, significant variability exists at every step of the process. This variability comes from sample collection and storage, DNA extraction methods, selection of amplification primers, sequencing technology, and handling computational elements used to quantify microbial communities [35]. Added to this is the lack of standardized protocols for handling microbial evidence, including guidelines for extraction, packaging, transport, and preservation. Additionally, the reliability of microbiome-based forensic tools needs significant improvement. Comprehensive studies are needed to establish error rates and assess reliability. Addressing these challenges will require large sample sizes and robust machine learning techniques, which are particularly well-suited for analyzing the complex, multidimensional data inherent to microbiomes [4]. All these challenges make the use of the microbiome difficult to use as evidence during a trial.

The findings suggest that microbial communities play a significant role in interactions, albeit with varying relative abundances depending on environmental factors such as wear, washing, and surface exposure. One of the most intriguing findings [25] is that worn and washed T-shirts retain similar microbial compositions at the phylum level, albeit with differences in relative abundance. This suggests that even after laundering, clothes continue to serve as long-term reservoirs of microbiome signatures from their wearers. The persistence of these microbial signatures for up to six months following wear presents an important consideration for forensic investigations, where clothes could be used to link individuals to specific environments or events long after physical contact has occurred.

In contrast, samples collected from glass surfaces [26] displayed reduced diversity in amplicon sequence variants (ASVs) compared to direct skin swabs. This indicates that the microbial communities transferred onto surfaces do not fully represent the original cutaneous microbiome, highlighting a critical challenge for forensic application. Given the reduced fidelity of transferred microbiota on surfaces, forensic comparisons between microbial traces found at a crime scene and a suspect’s skin microbiome must account for the diminished complexity of the surface-bound microbiota.

Moreover, the environmental persistence of human-associated bacteria, even after cleaning in controlled settings such as forensic laboratories [27], raises concerns about contamination. The observation of microbiome transfer onto personal protective equipment (PPE) and laboratory surfaces during mock examinations further underscores the need for strict procedural controls [28]. The presence of microbial communities on external surfaces of PPE, laboratory equipment, and cotton swatches following the examination suggests that microbiome transfer can occur during routine forensic processes, potentially complicating the interpretation of microbial evidence.

Another important finding is the successful detection of microbiome transfer between non-cohabitating individuals [29]. This reinforces the idea that microbial communities can be transferred through direct contact, though it also raises concerns regarding the specificity of such evidence. In a forensic context, microbial traces found at a crime scene may not necessarily indicate long-term proximity but could instead result from recent transient contact. This underlines the importance of contextual information when interpreting microbial evidence.

The dynamic nature of skin microbiota presents both opportunities and challenges for forensic applications. Another study [30] suggests that unlike fingerprints, which are stable over time, skin microbiota traces degrade rapidly, sometimes within hours, and are subject to temporal changes on both the host and the surface. This rapid degradation and variability make it difficult to equate microbial traces to the stability of traditional forensic evidence such as fingerprints. Timely collection of microbiome samples would thus be crucial for their utility in forensic investigations.

In built environments such as homes and offices, the microbial stability observed on frequently touched surfaces provides further insights into human–environment interactions. Areas with more frequent human contact, such as desks and nightstands, were more microbially similar to the occupants’ hands, whereas less frequently touched surfaces exhibited greater microbial diversity. This suggests that high-touch surfaces in a crime scene may yield more relevant forensic microbial data than low-touch or rarely accessed areas [31].

Interestingly, while roommates did not show significant convergence of their gut or skin microbiota over time, shared surfaces such as floors displayed a microbial composition reflective of both individuals [32]. This finding, along with the ability to predict an individual’s identity from the microbiota on their personal desk, indicates that microbial signatures are both highly individualized and spatially specific. The predictability of human-associated microbiota, even in shared spaces, highlights the potential for microbial traces to provide highly personalized forensic evidence.

Regarding the only article [33] focused on cadavers, it demonstrates that flies significantly influence the microbial communities involved in human decomposition, with the tarsi microbiome being the dominant contributor. The labellum microbiome plays a smaller role, while the oocyte microbiome contributes minimally or not at all under most conditions. However, warmer months alter these dynamics, leading to a decreased tarsi contribution and an increased role of the labellum. The oocyte microbiome also begins to contribute slightly in higher temperatures. These findings underscore the role of flies as vectors of microbes during decomposition and highlight the impact of environmental factors, such as temperature, on the microbial community assembly. This understanding can inform forensic science, particularly in improving postmortem interval estimations.

When discussing the microbiome, it is also important to consider the various environmental factors that alter its composition. Bacterial DNA is widely recognized for its greater resistance to degradation from UV light, heat, and humidity compared to human DNA. This resilience is due to its circular, condensed structure and the protection provided by a cell wall [36]. However, studies have shown that rising humidity and temperature levels generally promote the growth of skin bacteria. Research on the effects of low humidity on the skin microbiome is limited, although it is understood that bacterial growth tends to decrease in dry conditions [37].

This review also brings to light several challenges. The rapid degradation of microbial traces, the variability across different environments, and the risk of contamination during forensic procedures all pose significant hurdles to the reliable use of microbiome evidence. As the analogy between microbiota and fingerprints appears to be limited by the inherent temporal and spatial dynamics of microbial communities, the forensic application of microbiomes will require robust methodologies that account for these complexities. Furthermore, while national and international laws govern the study of the human microbiome for clinical and therapeutic purposes, there are no dedicated protocols or legal regulations for forensic applications. This can lead to difficulties in the interpretation of entomological, genetic, and medico-legal results. Above all, there is currently a lack of uniformity in the judicial decision-making of courts. Finally, there are other problematic issues pertaining to the forensic use of microbiomes: cost, training, and equipment in forensic laboratories [38].

### Study Limitations

This work has some limitations. The most important one is the small number of included articles. There are very few works investigating environmental transfer. In addition, we have encountered some difficulties in standardizing and synthesizing some data on materials as transfer vectors: each study used different materials to investigate the transfer. There is also a lack of uniformity regarding the environment used as the setting of the transfer.

Lastly, all the studies with one exception were conducted on living volunteers.

## 5. Conclusions

Interestingly, no article mentioned another significant place where the study of the microbiome could have extremely important forensic implications: the hospital [39]. This is a place where the occurrence of nosocomial infections or superinfections [40] can have an inauspicious outcome. It has been shown [41] how environmental microbes contribute to the transmission of pathogens in healthcare settings, underscoring the importance of microbial traceability evidence [42]. Furthermore, it is well established that surfaces and air in built environments play a significant role in microbial transfer, suggesting that unique microbial signatures can be used in forensic settings to trace human activity or presence [43]. In this very context, microbial resistance patterns may serve as forensic markers, particularly in hospital outbreaks [44].

While this study has demonstrated the potential for using microbiomes as trace evidence, particularly in linking individuals to environments or objects, the variability and dynamics of microbial transfer and persistence must be carefully considered. Future work should focus on developing standardized protocols for sampling [45], contamination control, and data interpretation to enhance the accuracy and reliability of microbiome evidence in medico-legal investigations. When performing analysis on microbiome, it is essential for specialists from a wide range of fields—such as legal medicine, microbiology, ecology, and bioinformatics—to work together to create best practices. This collaboration helps identify and address sources of potential variability that can impact measurement accuracy. Reducing variability is critical to developing optimal research protocols and for allowing the integration of results from multiple studies into larger analyses.

Using microbiome data in forensics is challenging due to variability in sample collection, DNA extraction, and analysis methods, along with a lack of standard protocols for handling microbial evidence. Reliability remains a concern, as robust studies and advanced machine learning are needed to address these complexities, making microbiome evidence difficult to reliably present in legal cases.

Finally, it is interesting to note that there are numerous clinical studies and meta-analyses on the microbiome [46], which provide interesting insights. However, in the forensic field, despite the presence of numerous systematic reviews published on this topic, there are none that focus on its environmental transfer alone. This requires conducting more forensic studies in order to have sufficient data to conduct relevant meta-analyses.

## Figures and Tables

**Table 1:** Synthesis table of the studies analyzed.

Living				
Authors and Year of Publication	Country of Affiliation	Number of Samples	Mode of Transfer	Main Findings
Procopio N et al. (2024) [25]	United Kingdom and Italy	2 volunteers	Participants wore freshly washed clothes for 24 h. Samples were taken from the neck area of the T-shirt and analyzed to assess the transfer of microbes from the skin of the neck to the T-shirts.	The results indicate that both the skin microbiome and the textile microbiome are predominantly composed of three phyla (Firmicutes, Actinobacteria, and Proteobacteria), which exhibit varying relative abundances depending on whether the T-shirt has been worn. Interestingly, the analysis of a clean shirt after washing revealed a similar microbial composition at the phylum level compared to the worn T-shirt, albeit with different relative abundances. The article also suggests that the microbiome transferred from the skin to the clothing can be reliably sampled for up to six months following the transfer.
Procopio N et al. (2021) [26]	United Kingdom and Italy	11 volunteers	Samples were collected by gently sliding two sterile swabs moistened with physiological saline over the entire surface of the palm, including the fingers, of the dominant hand for 15 s. The same participants were then instructed to touch two glass microscope slides with all five fingers for approximately 10 s to leave their fingerprints across the surface. After 30 days at room temperature, the deposited fingerprints were swabbed to collect a “glass fingerprint sample.”	Samples taken from a glass surface exhibited a lower number of amplicon sequence variants (ASVs) compared to the skin swab samples, indicating that the microbial fingerprint transferred does not completely reflect the skin microbiome. When comparing a microbial trace found at a crime scene to the skin microbiome of a potential suspect, it is essential to obtain a similar type of trace to that found on-site (for example, a fingerprint left on a glass slide).
Neckovic A. et al. (2021) [27]	Australia	31 swabs obtained in an evidence recovery laboratory (ERL)	Multiple surfaces of the ERL were swabbed before a monthly deep cleaning, immediately after the cleaning, and again after one day of use by a participant who had employed the ERL for several routine item examinations.	None of the samples collected from the ERL surfaces exhibited any consistent microbial signatures over time, indicating that the surfaces were likely influenced more by contributions from the human microbiome or by activity-related disruptions and contributions (such as cleaning and item examinations). While samples from built environments are frequently characterized as low biomass, meaning they may be affected by sampling efforts, the background microbiomes present in forensic settings might not be distinguishable from the target microbial communities associated with forensic evidence.
Neckovic A. et al. (2021) [28]	Australia	Three volunteers (86 samples)	Swatches of unused, non-sterile, white 100% cotton were rubbed vigorously and separately between the hands of a volunteer for 10 s each, ensuring even contact. The cotton swatches were then placed in a plastic zip-lock bag and sealed at room temperature for five days. To identify the occurrence and potential sources of microbial transfer during this process, samples were taken from the external surfaces of worn personal protective equipment (PPE), various laboratory surfaces, and equipment used during the mock examinations, both before and after use by each participant.	For participant three, microbiome transfer was detected in several samples collected from the external surfaces of the PPE and the laboratory, including the examined cotton swatch. In contrast, for participants one and two, this microbiome transfer appeared to be less extensive across the samples; however, it was still present in the profiles of the external surfaces of their PPE and laboratory samples, as well as in the examined cotton swatch for participant one. Thus, it cannot be ruled out that the microbial communities observed in the sample profiles of the external surfaces of the PPE, laboratory surfaces and equipment (after examination), and the examined cotton swatches resulted from physical microbiome transfer during the mock examination and subsequent sampling.
Neckovic A. et al. (2020) [29]	Australia	3 pairs of volunteers (65 samples)	Mode 1: 1.1 A firm, 30 s handshake between participants. 1.2 Microbiome transfer from the right hand onto a single surface following the handshake. Mode 2: Microbiome transfer from the left hand onto a single surface for 30 s, after which participant pairs exchange cotton or paper swatches or glass marbles and repeat the transfer process for another 30 s.	This preliminary study has demonstrated that skin microbiome transfer can occur between individuals who do not live together, across the specified transfer methods and substrate types used. This finding highlights the potential of microbiomes as trace evidence in investigations. The data suggest that microbial communities exchanged through direct skin contact may influence the microbial composition of a profile generated from a particular body site sampled from an individual.
Wilkins et al. (2017) [30]	China	812 samples (144 air samples, skin samples 380, surface samples 288)	Microbiota samples collected from household surfaces, indoor air, and the skin of residents across nine homes.	When skin and surface samples collected simultaneously were analyzed, the correct occupant or occupants were accurately identified in 67% of cases. Human skin microbiota shifts over time, and microbial traces left on surfaces begin to degrade within hours, even without cleaning or other physical disturbance. This study indicates that comparing microbiota traces to fingerprints can be misleading; unlike fingerprints, skin microbiota evolves over time on both the host and on surfaces where traces are left.
Hoisington et al. (2023) [31]	United States of America	426 samples (252 office samples, 174 home samples)	Each of the 22 participants occupied a private office. For three consecutive weeks, an investigator swabbed each participant’s dominant palm, entire computer mouse, entire keyboard, and a one square-foot section of their desk on a weekly basis. Approximately one month after the office sampling concluded, a subset of participants (n = 11) began home sampling. For three consecutive weeks, these participants self-collected samples from their dominant palm, bedroom nightstand, bathroom counter, bedroom floor, and living room floor. Additionally, when present, the adult partners of participants self-sampled their dominant palm (n = 6).	This study found microbiota stability on both the hands and in the built environment within office and home settings; however, stability did not extend consistently between these two environments. Surfaces and objects in frequent contact with occupants were more similar, microbially, to the occupants’ hands than those with less frequent contact. The extent to which occupants shared their microbial community with the built environment varied among participants, and horizontal surfaces (such as desks, nightstands, and living room floors) showed greater microbial diversity compared to objects or occupants’ hands. Microbes from both human hands and environmental surfaces at each location remained relatively stable over the 3-week sampling period.
Sharma et al. (2019) [32]	United States of America	2170 samples	34 United States Air Force Academy cadets occupying 21 rooms	Although roommates did not show a significant increase in the similarity of their gut or skin microbiota over time, they remained significantly more like each other than to non-roommates. The microbiota on each desk closely matched the cadet who used it, more so than any other cadet, while the shared floor space between beds was more like both roommates than to any other cadet. The bacterial profile associated with everyone was highly predictive of their identity, with the gut microbiome being more distinct than the more variable skin microbiota. Within a dormitory room, the desk microbiota could reliably identify the cadet who regularly interacted with it, nearly as accurately as the cadet’s own gut microbiota. The floor area between beds could correctly identify the two cadets who lived in the room with over 80% accuracy.
**Cadaveric**				
**Authors and Year of Publication**	**Country of Affiliation**	**Number of Samples**	**Mode of Transfer**	**Conclusions**
Deel H.L. et al. (2022) [33]	United States of America	10 cadavers and the first 40 flies that were in contact with each cadaver	Direct transfer from the flies to the cadavers	The tarsi microbiome is a major contributor to the human decomposition microbiome, while the labellum microbiome plays a smaller role, and the oocyte microbiome contributes minimally. As temperatures rise in warmer months, the proportion from the labellum increases, the tarsi contribution decreases, and the oocyte microbiome begins to play a slight role in the decomposition microbiome. This study provides evidence that microbial transfer from flies to humans during decomposition influences the assembly of the microbial community on human cadavers.

## Supplementary Materials

The following supporting information can be downloaded at: https://www.mdpi.com/article/10.3390/microorganisms12122424/s1, Table S1: PRISMA 2020 Main Checklist.
